# Metyrapone Treatment Protects Low-Density Lipoprotein Receptor Knockout Mice against Hypercorticosteronemia Development without Changing Atherosclerosis Susceptibility

**DOI:** 10.3390/biom13091287

**Published:** 2023-08-23

**Authors:** Ronald J. van der Sluis, Tim van den Aardweg, Timothy J. P. Sijsenaar, Miranda Van Eck, Menno Hoekstra

**Affiliations:** 1 Division of BioTherapeutics, Leiden Academic Centre for Drug Research, Leiden University, 2333CC Leiden, The Netherlands; r.j.van_der_sluis@lumc.nl (R.J.v.d.S.); m.eck@lacdr.leidenuniv.nl (M.V.E.); 2 Division of Systems Pharmacology and Pharmacy, Leiden Academic Centre for Drug Research, Leiden University, 2333CC Leiden, The Netherlands; 3 Pharmacy Leiden, Leiden, The Netherlands

**Keywords:** glucocorticoid, corticosterone, metyrapone, 11beta-hydroxylase, atherosclerosis, LDL receptor knockout mice, lymphocytopenia

## Abstract

The steroid 11beta-hydroxylase inhibitor metyrapone is able to effectively reverse the hypercortisolemia detected in human Cushing’s Syndrome patients. In this current preclinical study, we investigated whether metyrapone monotherapy can also reverse the hypercortisolemia-associated increase in atherosclerotic cardiovascular disease risk. In this instance, female low-density lipoprotein receptor knockout mice fed a cholic acid-containing high cholesterol/high fat diet to induce the development of hypercorticosteronemia and atherosclerotic lesions were treated twice daily with 100 mg/kg metyrapone for 4 weeks. Metyrapone effectively protected against hypercorticosteronemia development with endpoint plasma corticosterone levels remaining 43% lower than in controls (*p* < 0.01). Gene expression analysis in livers and adrenals validated that glucocorticoid receptor signaling was also reduced. Importantly, metyrapone treatment did not impact plasma cholesterol levels or alter atherosclerotic plaque areas or lesional collagen contents. However, metyrapone induced significant systemic lymphocytopenia as evident from marked decreases in splenic white pulp contents and thymus weights (−48% and −41%, respectively; *p* < 0.001). In conclusion, we have shown that treatment with metyrapone diminishes hypercorticosteronemia without affecting atherosclerosis susceptibility in cholic acid-containing high cholesterol/high fat diet-fed low-density lipoprotein receptor knockout mice. These preclinical findings highlight that restoring plasma glucocorticoid levels to normal is not necessarily sufficient to overcome the cardiovascular co-morbidities associated with human Cushing’s disease.

## 1. Introduction

Cushing’s Syndrome is a rare pathology characterized by prolonged exposure to glucocorticoid hormones such as cortisol due to endogenous overproduction by the adrenals or an excessive intake of cortisol-like medication such as prednisone. A meta-analysis by Lupoli et al. [1] has identified that subjects suffering from Cushing’s Syndrome, as compared to unaffected human individuals, exhibit a relatively high prevalence of carotid atherosclerotic plaques and an increased intima-media thickness which is a surrogate marker of atherosclerosis burden. In accordance, Etxabe and Vazquez have observed a fivefold higher cardiovascular disease standardized mortality rate in Cushing’s disease patients as compared to subjects from the general population [2]. Since an increase in serum and urinary cortisol levels is positively associated with higher common carotid artery intima-media thickness [1], it can be hypothesized that hypercortisolemia is the driving force behind the increased atherosclerosis susceptibility of Cushing’s Syndrome patients.

Interestingly, a multi-center trial executed by Daniel et al. has shown that pharmacological inhibition of the cortisol-generating enzyme steroid 11beta-hydroxylase (CYP11B1) through chronic treatment with metyrapone is able to bring relief to the majority of Cushing’s disease patients from their hypercortisolemia [3]. To date, it remains unknown what the effect is of chronic metyrapone monotherapy on Cushing’s disease-associated atherosclerotic cardiovascular disease risk in humans. We therefore investigated the effect of chronic metyrapone treatment on atherosclerosis susceptibility in a Cushing’s Syndrome-like preclinical setting.

## 2. Materials and Methods

### 2.1. Mice and Treatment

Animal experiments were performed in a temperature and light cycle (12 h light/12 h dark) controlled room at the Gorlaeus Laboratories of the Leiden Academic Centre for Drug Research, Leiden University in accordance with the National Laws and the ARRIVE guidelines. Protocols were approved by the Ethics Committee for Animal Experiments of Leiden University. Group-housed low-density lipoprotein (LDL) receptor knockout mice (10–12 weeks old; Jackson Laboratories) were challenged with a diet containing 15% (*w*/*w*) fat, 1% (*w*/*w*) cholesterol, and 0.5% (*w*/*w*) cholic acid (Abdiets) to stimulate the development of atherosclerotic lesions. Female LDL receptor knockout mice were used as they generally display a higher susceptibility to high fat/high cholesterol diet-induced atherosclerotic lesion development as compared to their male counterparts [4,5], and the incidence of Cushing’s Syndrome in humans has been shown to be three times higher in females than in males [6]. The experimental group of mice (N = 11) was administered metyrapone (CAS Number: 54-36-4; Sigma-Aldrich, Amsterdam, The Netherlands) intraperitoneally twice daily at a dose of 100 mg/kg body weight, whilst the age- and body-weight matched control group (N = 12) received similar injections with the solvent control PBS (dosing volume: 100 µL). The injections were given alternately at the left and right side of the abdomen to minimize tissue damage and scar formation. The total daily dose of 200 mg/kg of metyrapone was based upon the dose of 1375 mg per day that has been shown to be effective in reducing cortisol levels in human Cushing’s disease patients [3], while also talking into account the 10 times higher metabolic rate in mice as compared to humans. After four weeks of atherogenic diet feeding and parallel injections, mice were anesthetized and sacrificed through injection of a mix of xylazine, ketamine, and atropine after overnight food deprivation. After whole body perfusion with PBS, the heart, adrenals, liver, thymus, and spleen were isolated and stored in 3.7% formaldehyde or at −20 °C until further use.

### 2.2. Plasma Analysis

Blood was drawn from the tail of the mice into Sarstedt microvette CB300 EDTA spiked tubes before and after the atherogenic diet challenge within 30 s from the initial mouse handling to measure levels of the primary glucocorticoid species corticosterone in plasma using an ^125^I radioimmunoassay (MP Biomedicals, Illkirch Cedex, France) according to the manufacturer’s instructions. The concentration of total cholesterol in plasma samples, collected from the retro orbital sinusat sacrifice, were determined by enzymatic colorimetric assays with 0.025 U/mL cholesterol oxidase (Sigma-Aldrich), 0.065 U/mL peroxidase (Roche Diagnostics, Almere, The Netherlands), and 15 μg/mL cholesteryl esterase (Roche Diagnostics) in reaction buffer (1.0 mol/L KPi buffer, pH = 7.7 containing 0.01 mol/L phenol, 1 mmol/L 4-amino-antipyrine, 1% polyoxyethylene-9-laurylether, and 7.5% methanol). Absorbance was read at 490 nm. Triglycerides were quantified in eye blood plasma samples using a kit from Roche Diagnostics. Precipath (standardized serum; Roche Diagnostics) was used as internal standard for both the cholesterol and triglyceride measurements.

### 2.3. Hematological Analysis

The concentration of platelets, red blood cells, and white blood cells in sacrifice blood samples and the distribution of the white blood cells over the different leukocyte subclasses were routinely determined using a Sysmex XT-2000i Hematology Analyzer (Sysmex, Etten-Leur, The Netherlands).

### 2.4. Immunohistochemistry

Cryosections of spleens (8 μm) and hearts (10 μm) were prepared on a Leica CM 3050s cryostat (Leica microsystems, Amsterdam, The Netherlands). Spleen sections were stained with hematoxylin to identify white pulp areas. Images of the spleens were obtained using a Leica DMRE microscope connected to a Leica DC 500 camera (Leica microsystems). Relative white pulp areas of six mice randomly chosen from each group were quantified using Leica Qwin standard v3 software (Leica microsystems). Heart cryosections where the three-valve area was visible were stained with Oil Red O (Sigma-Aldrich) or Masson’s Trichrome (Sigma-Aldrich) to subsequently quantify neutral lipid-containing areas and the intraluminal lesion areas as well as the plaque collagen contents, respectively. Two hearts from each experimental group were lost during embedding and sectioning and could therefore not be included in the atherosclerotic lesion analysis. Five aortic root sections per individual mouse were analyzed. All quantifications were performed blinded.

### 2.5. Gene Expression Analysis

Total RNA was isolated from livers and adrenals from five to seven randomly chosen mice per group using a phenol/chloroform extraction method and subsequently reverse transcribed. Gene expression analysis was performed using real-time SYBR Green technology (Eurogentec, Seraing, France), as described [7]. Beta-actin (ACTB), glyceraldehyde-3-phosphate dehydrogenase (GAPDH), ribosomal protein, and large, P0 (36B4) were used as the standard housekeeping genes.

### 2.6. Data Analysis

Statistical analysis was performed using Graphpad Prism Software version 8.0. Normality testing of the experimental groups was performed using the method of Kolmogorov and Smirnov. A Grubb’s test was executed to detect potential outliers. Significance was calculated using a two-tailed *t*-test or two-way analysis of variance (ANOVA) with a Bonferroni post-test. Probability values less than 0.05 were considered significant.

## 3. Results

**Figure 1:**
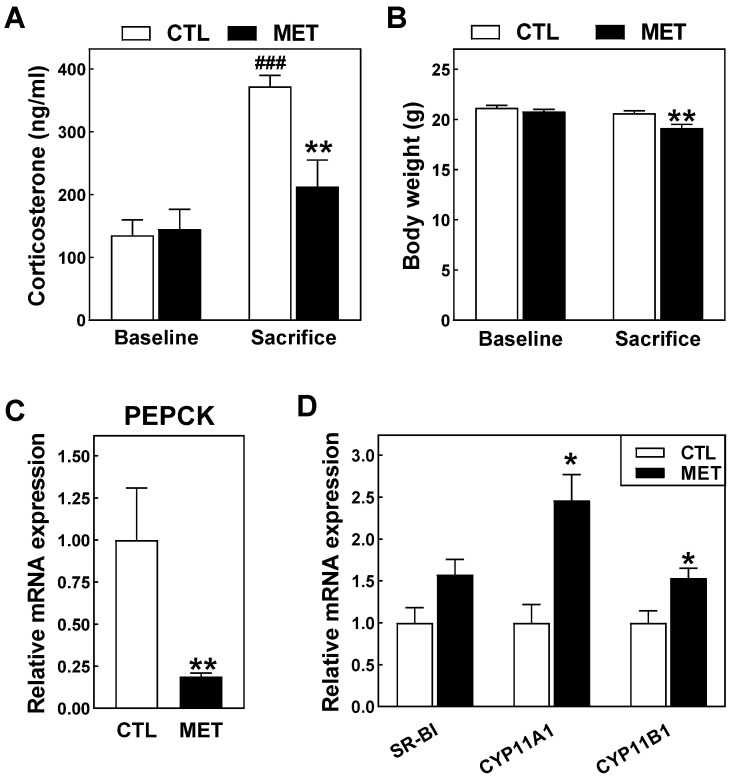
Plasma corticosterone levels (**A**), body weights (**B**), hepatic PEPCK gene expression levels (**C**), and adrenal relative mRNA expression levels (**D**) in atherogenic diet-fed female LDL receptor knockout mice injected two times per day with 100 mg/kg metyrapone (MET; black bars) or solvent control PBS (CTL; white bars) for 4 weeks. Data are presented as means + SEM for 11 metyrapone-treated and 12 control mice per group (panels **A**,**B**) or 5–7 mice per group (panels **C**,**D**). ### *p* < 0.01 versus Baseline. * *p* < 0.05, ** *p* < 0.01 versus CTL.

To evaluate the potential impact of metyrapone treatment on atherosclerosis susceptibility, we made use of genetically hypercholesterolemic LDL receptor knockout mice that were fed an atherogenic high cholesterol/high fat Western-type diet also containing the farnesoid X receptor (FXR) ligand cholic acid. Our previous studies have shown that FXR activation increases the adrenal glucocorticoid output [8], and that chronic cholic acid-containing diet feeding is therefore associated with Cushing’s-like hypercorticosteronemia induction in LDL receptor knockout mice [9]. In accordance, four weeks of atherogenic diet feeding was associated with a 2.8-fold rise (*p* < 0.001) in plasma corticosterone levels in control-treated LDL receptor knockout mice (Figure 1A). In contrast, a non-significant 1.5-fold increase was detected in plasma corticosterone concentrations in atherogenic diet-fed LDL receptor knockout mice that were administered twice daily with a dose of 100 mg/kg metyrapone. As a result, metyrapone-treated LDL receptor knockout mice exhibited 43% lower (*p* < 0.01) plasma corticosterone levels as compared to those in PBS-injected controls at the final 4-week time point (Figure 1A). Ceccato et al. have previously observed that metyrapone-mediated reversal of hypercortisolemia is paralleled by a reduction in body weight in human patients with Cushing’s Syndrome [10]. In accordance, significant weight loss was detected only in the group of atherogenic diet-fed LDL receptor knockout mice treated with metyrapone (−8% versus Baseline; *p* < 0.01; Figure 1B). No metyrapone treatment-associated change was observed in adrenal weights (15 ± 4 mg for metyrapone-treated mice versus 15 ± 3 mg for controls; *p* > 0.05). Glucocorticoids such as cortisol and corticosterone induce their biological effects, i.e., stimulation of gluconeogenesis and immunosuppression, through activation of the nuclear transcription factor glucocorticoid receptor (GR) [11]. Gene expression analysis on isolated livers and adrenals verified that the pharmacological inhibition of CYP11B1 activity by metyrapone treatment did not only significantly lower plasma corticosterone levels, but also translated into a diminished tissue GR activity. More specifically, metyrapone treatment reduced hepatic relative mRNA expression levels of the gluconeogenic GR target gene phosphoenolpyruvate carboxykinase (PEPCK; −81%; *p* < 0.01; Figure 1C). In addition, metyrapone treatment was associated with increases in adrenal relative mRNA expression levels of the high-density lipoprotein receptor SR-BI (+58%; *p* = 0.07) and the steroidogenic enzymes CYP11A1 (+146%; *p* < 0.05) and CYP11B (+53%; *p* < 0.05) (Figure 1D), whose gene expression is subject to negative feedback regulation in response to GR activation [12,13].

**Figure 2:**
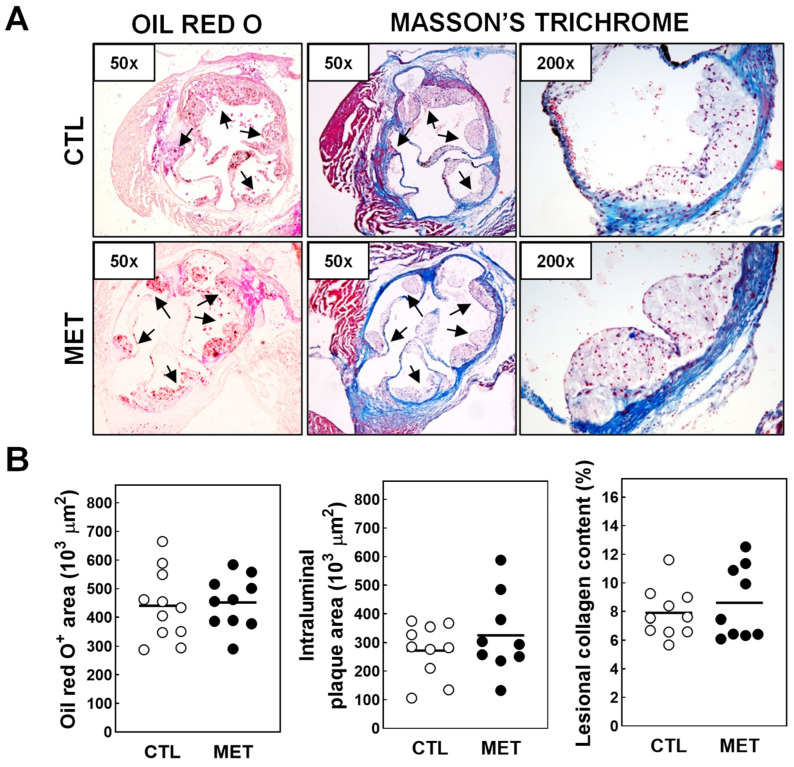
(**A**) Representative images of cryosections showing the presence of atherosclerotic lesions in the aortic root of both groups of mice. The original magnification of each section image is provided in the upper left-hand corner. Arrows points towards atherosclerotic lesions. (**B**) Quantifications of the Oil Red O-positive neutral lipid areas, intraluminal plaque areas, and lesional collagen contents in atherogenic diet-fed female LDL receptor knockout mice injected two times per day with 100 mg/kg metyrapone (MET; black dots) or solvent control PBS (CTL; white dots) for 4 weeks. Data are presented as individual mouse data points with the group averages shown as horizontal lines.

In line with the notion that four weeks of atherogenic diet feeding will be associated with initial atherosclerotic lesion development, no plaques were microscopically visible in the aortic arch of any of our female LDL receptor knockout mice. As such, aortic arches or the ascending part of the aorta were not isolated from the mice during sacrifice. However, as can be appreciated from the representative images of the Oil Red O- and Masson’s Trichome-stained three valve heart cryosections in Figure 2A, atherosclerotic lesions could be detected in the aortic root in both experimental groups of mice. Lesions were primarily of an early-stage type as judged from the observation that they mostly consisted of Oil Red O-positive neutral lipid deposit-containing cells, i.e., macrophage foam cells, were essentially devoid of smooth muscle cells, and contained limited amounts of collagen. Strikingly, quantification of Oil Red O-positive areas revealed that metyrapone treatment did not significantly change the atherosclerosis susceptibility (Figure 2B). In further support of a virtually unaffected atherosclerosis susceptibility, intraluminal plaque areas as well as lesional collagen contents were also similar in metyrapone-treated and control LDL receptor knockout mice (Figure 2B).

**Figure 3:**
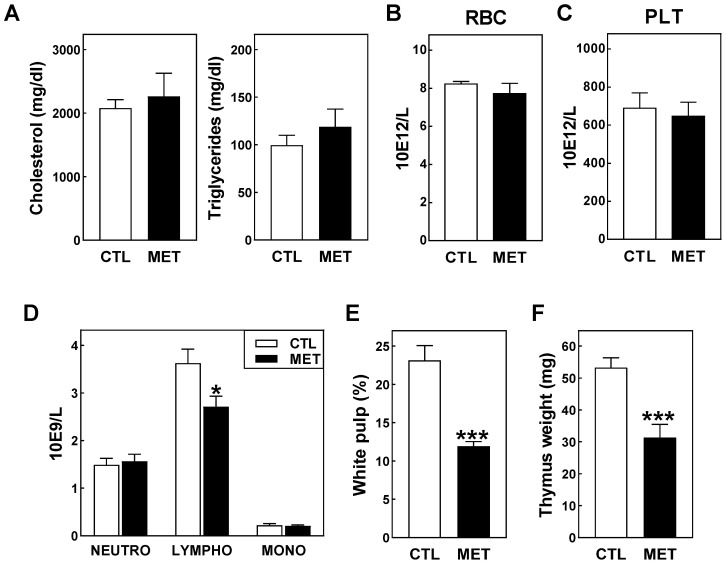
Plasma lipid levels (**A**), blood erythrocyte/red blood cell (**B**), platelet (**C**), and neutrophil, lymphocyte, and monocyte (**D**) concentrations, splenic white pulp fractions (**E**), and thymus weights (**F**) in atherogenic diet-fed female LDL receptor knockout mice injected two times per day with 100 mg/kg metyrapone (MET; black bars) or the solvent control PBS (CTL; white bars) for 4 weeks. Data are presented as means + SEM of 10/11 metyrapone-treated and 11/12 control-treated mice per group. * *p* < 0.05, *** *p* < 0.001 versus CTL.

Atherosclerosis susceptibility is dependent on the accumulation of pro-atherogenic lipid particles in the plasma compartment as well as on the (systemic) immune reaction to the pathological lipid deposition within the vessel wall [14]. We therefore investigated whether chronic metyrapone treatment impacted plasma lipid levels or the hematological/systemic immune profile. As can be seen from Figure 3A, no change in the hyperlipidemia extent was detected in response to metyrapone treatment. Both plasma cholesterol and triglyceride levels were not significantly different between metyrapone-treated and control LDL receptor knockout mice after 4 weeks of atherogenic diet feeding. Metyrapone treatment also did not modify blood concentrations of erythrocytes/red blood cells (RBC) and platelets (PLT) (Figure 3B,C). However, white blood cell (WBC) numbers were found to be significantly decreased in response to metyrapone exposure (4.6 ± 0.3 × 10^9^/L for metyrapone-treated mice versus 5.6 ± 0.3 × 10^9^/L for control-treated mice, respectively; *p* < 0.05). Since blood concentrations of neutrophils and monocytes were not different between the two experimental groups (Figure 3D), the decrease in total leukocyte counts found in metyrapone-treated mice could be solely attributed to a 25% reduction in circulating lymphocytes (*p* < 0.05; Figure 3D). Quantification of the relative area occupied by white pulp revealed that splenic lymphocyte numbers were also reduced upon metyrapone treatment (−48%; *p* < 0.001; Figure 3E). Notably, these effects were paralleled by a 41% reduction in the weight of the thymus which is the primary lymphoid organ that controls the maturation of both cytotoxic and helper T cells (*p* < 0.001; Figure 3F).

## 4. Discussion

Here we tested in a preclinical mouse model the hypothesis that the hypercortisolemia in Cushing’s Syndrome patients is the driving force behind the associated higher atherosclerotic cardiovascular disease risk and that reversal of the hypercortisolemia through metyrapone treatment will thus also lead to a reduction in atherosclerosis susceptibility. We have found that exposure to a clinically relevant dose of metyrapone does effectively protect hyperlipidemic LDL receptor knockout mice against hypercorticosteronemia development in response to cholic acid-containing atherogenic high cholesterol/high fat diet feeding. In contrast, chronic metyrapone treatment did not also reduce the susceptibility to diet-induced atherosclerotic lesion development.

Hypercholesterolemia is considered a driving force in the development of atherosclerotic lesions in LDL receptor knockout mice [15]. The observation that the lowering of plasma glucocorticoid levels and GR signaling through metyrapone treatment did not also reduce the hyperlipidemia extent may thus perhaps underlie the unexpected overall null effect on atherosclerosis outcome. However, it should be appreciated that the increased atherosclerotic cardiovascular disease risk in Cushing’s patients does not seem to be solely related to exacerbated hypercholesterolemia. Sun et al. [16] have found that only 36.4% of hypercortisolemic Cushing’s Disease patients actually exhibit hyperlipidemia, whilst >70% of these individuals also suffer from hypertension and impaired glucose metabolism which are two established independent atherosclerotic cardiovascular disease risk enhancers [17,18]. Normalizing blood pressure and glucose tolerance may thus be even more effective than reducing the hypercholesterolemia extent in order to reverse the enhanced cardiovascular disease risk in Cushing’s Syndrome patients. Unfortunately, we did not measure blood pressure or determine the glucose tolerance in our different experimental mouse groups. Bencze et al. have previously found that metyrapone-mediated suppression of endogenous glucocorticoid synthesis reduces the blood pressure and heart rate response to restraint in rats [19]. As such, it can be assumed that metyrapone will be able to restore blood pressure levels to normal also in a Cushing’s Syndrome setting. Based upon our observation that metyrapone treatment markedly reduced the hepatic gene expression levels of the gluconeogenic enzyme PEPCK, we anticipate that metyrapone will also be able to functionally restore glucose homeostasis. However, it should be noted that lower dosages of metyrapone than used in our current study, i.e., 30 mg/kg and 50 mg/kg versus 100 mg/kg, were not sufficient to reverse the hyperglycemic effect of the 5HT_1A_ agonist 8-hydroxy-2-(di-n-propylamino)tetralin in normolipidemic C57BL/6 wild-type mice [20]. Dedicated follow-up studies are therefore warranted to uncover the actual effect of higher metyrapone dosages on the systemic glucose tolerance in a Cushing’s Syndrome setting as the inability of metyrapone to normalize glucose tolerance may still potentially explain our observed null effect on atherosclerosis susceptibility. Another potential explanation for the lack of an effect on atherosclerosis outcome in our current study is that enhanced secretion of adrenal-derived steroid metabolites other than corticosterone, i.e., 11-deoxycorticosterone, has nullified the beneficial impact of the metyrapone-mediated hypercorticosteronemia ablation. Raven et al. have shown that, in a small cohort of mentally depressed human subjects, metyrapone treatment was associated with a striking >5-fold increase in the urinary output of 11-deoxycortisol (deoxycortisol) metabolites [21]. Treatment with deoxycorticosterone stimulates atherogenesis in Watanabe heritable hyperlipidemic rabbits [22] as well as in hyperlipidemic apolipoprotein E knockout mice [23]. Lother et al. have shown that deoxycorticosterone executes its pathological vascular effects through activation of the mineralocorticoid receptor (MR) [24]. It can therefore be hypothesized that treatment with a combination of metyrapone and a potent MR antagonist such as finerenone, spironolactone, or eplerenone [25] may be able to achieve a significant reduction in Cushing’s Syndrome-associated atherosclerosis susceptibility.

An interesting finding of our study is that metyrapone treatment induced a global decrease in lymphocyte numbers. A systemic decrease in glucocorticoid functioning due to bilateral adrenalectomy is usually associated with a rise in blood lymphocyte counts [9,26]. The occurrence of lymphocytopenia in response to metyrapone treatment-induced lowering of plasma glucocorticoid levels can therefore be perceived as unexpected. Transcript levels of CYP11B1 were absent in the spleens of both of our mice groups, which argues against a direct effect of metyrapone on lymphocytes in this compartment. In contrast, Vacchio et al. have shown that in vitro exposure of primary murine thymocytes to metyrapone results in a marked loss of CD4/CD8 double positive subtypes, accommodated by an enhanced apoptosis rate due to a diminished positive selection [27,28]. CYP11B1 mRNA expression can be detected, albeit at low levels, within the thymus [29]. In light of the significant decrease in thymus weight detected upon metyrapone treatment, it is suggested that glucocorticoids produced locally within the thymus control positive T cell selection and that metyrapone inhibits this process to eventually lower total body T cell numbers.

In conclusion, we have shown that treatment with metyrapone diminishes hypercorticosteronemia without affecting atherosclerosis susceptibility in cholic acid-containing high cholesterol/high fat diet-fed LDL receptor knockout mice. Our preclinical findings highlight that restoring plasma glucocorticoid levels to normal is not necessarily sufficient to overcome the cardiovascular co-morbidities associated with human Cushing’s disease. In addition, our findings warrant further in-depth studies on the potential (negative) impact of chronic metyrapone treatment on human immunity.

## Data Availability

All data associated with this study are available from the corresponding author upon reasonable request.
